# Role of Cytosolic 2-Cys Prx1 and Prx2 in Redox Signaling

**DOI:** 10.3390/antiox8060169

**Published:** 2019-06-10

**Authors:** Yosup Kim, Ho Hee Jang

**Affiliations:** 1 Department of Health Sciences and Technology, Graduate School of Medicine, Gachon University, Incheon 21999, Korea; youandkys@naver.com; 2 Department of Biochemistry, College of Medicine, Gachon University, Incheon 21999, Korea

**Keywords:** peroxiredoxin, reactive oxygen species, hyperoxidation, antioxidant enzymes, peroxidase activity, chaperone activity, protein-protein interaction

## Abstract

Peroxiredoxins (Prxs), a family of peroxidases, are reactive oxygen species scavengers that hydrolyze H_2_O_2_ through catalytic cysteine. Mammalian Prxs comprise six isoforms (typical 2-Cys Prxs; Prx1–4, atypical 2-Cys Prx; Prx5, and 1-Cys Prx; Prx6) that are distributed over various cellular compartments as they are classified according to the position and number of conserved cysteine. 2-Cys Prx1 and Prx2 are abundant proteins that are ubiquitously expressed mainly in the cytosol, and over 90% of their amino acid sequences are homologous. Prx1 and Prx2 protect cells from ROS-mediated oxidative stress through the elimination of H_2_O_2_ and regulate cellular signaling through redox-dependent mechanism. In addition, Prx1 and Prx2 are able to bind to a diversity of interaction partners to regulate other various cellular processes in cancer (i.e., regulation of the protein redox status, cell growth, apoptosis, and tumorigenesis). Thus, Prx1 and Prx2 can be potential therapeutic targets and it is particularly important to control their level or activity. This review focuses on cytosolic 2-Cys Prx1 and Prx2 and their role in the regulation of redox signaling based on protein-protein interaction.

## 1. Introduction

Cells are constantly exposed to various forms of extra- or intracellular stress, and such stress continuously leads to the production of reactive oxygen species (ROS) including radical or non-radical oxygen species [1]. The types of ROS include superoxide radical, hydrogen peroxide, hydroxyl radical, and singlet oxygen [2]. A low concentration of ROS acts as a second messenger, playing an important part in normal cellular growth and function whereby cellular redox signaling and homeostasis are regulated [3]. However, a high concentration of ROS, apart from being toxic and harmful to the cells, induces an imbalance of ROS regulation that acts as oxidative stress, becoming a cause of various diseases [1,4]. As it is of utmost importance to remove the increased ROS, cells have a defense system for effective ROS removal [5]. The most well-known oxidative stress defense system is the antioxidant enzyme. The antioxidant enzymes are superoxide dismutase (SOD), catalase (CAT), glutathione peroxidase (GPx), and peroxiredoxin (Prx) [6,7,8,9]. Among them, the Prxs family does not rely on a cofactor that other antioxidant enzymes use but on one or two conserved active cysteine (Cys) to mediate a Cys-dependent reduction of intracellular H_2_O_2_ [10].

Mammalian Prxs comprise six isoforms. According to the position and number of conserved Cys, they are classified into typical 2-Cys Prxs (Prx1–4), atypical 2-Cys Prx (Prx5), and 1-Cys Prx (Prx6) [11]. Prx1 and Prx2 are mostly expressed abundantly in the cytosol, although some are observed in the nucleus [12,13]. When low levels of H_2_O_2_ cause the oxidation of Prx1 and Prx2 into a sulfenic form (Cys-SOH) to form intermolecular disulfide bond with other subunits of Prx, they are reduced back via the thioredoxin (Trx)/Trx reductase (TrxR) system for effective removal of intracellular ROS. Nonetheless, high levels of H_2_O_2_ can cause the hyperoxidation of Prx1 and Prx2 into a sulfinic form (Cys-SO_2_H). The resulting Prx-sulfinic form may undergo reversible reduction by sulfiredoxin (Srx), but the reduction by Srx is extremely slow compared to Trx while the activity of Srx is also low [14]. The persistent state of high H_2_O_2_ levels transform Prx1 and Prx2 into an irreversible hyperoxidized form (Prx-sulfonic acid). In addition, the generation of the hyperoxidized forms of Prx1 and Prx2 induce a structural change to high-molecular-weight (HMW) complexes, with a functional switch as their peroxidase activity disappears and chaperone activity gets acquired [15,16].

Prx1 and Prx2 exhibit not only peroxidase and chaperone activities, but also an ability to interact with other proteins to participate in diverse intracellular processes such as cell growth, apoptosis, and carcinogenesis, and neuronal differentiation [17,18,19]. This review describes the various antioxidant enzymes and focuses on the cytosolic 2-Cys Prx subtypes Prx1 and Prx2 that are the most abundant among mammalian Prxs family and how they regulate their interacting protein partners to participate in redox signaling in the light of cancer signaling.

## 2. ROS, Oxidative Stress and Cancer

ROS is an oxygen byproduct produced by the aerobic metabolism of cells [20]. It refers to the unstable oxygen species with unpaired electrons that are also called free radicals [21]. Cells also produce ROS from diverse extracellular and intracellular sources. The most representative intracellular sources that lead to ROS are nicotinamide adenine dinucleotide phosphate (NADPH) oxidase, xanthine oxidase, and the mitochondria [22]. Among them, the electron transport chain of the mitochondria is the major source, producing over 80–90% of ROS. The relatively stable O_2_ is mostly reduced to water directly during aerobic respiration [23].

O_2_ + 4e^−^ + 4H^+^ → 2H_2_O (1)

However, continuous influx of electrons for the consecutive reduction of O_2_ leads to the increase in various types of ROS. The most well-known ROS produced in this way are superoxide anion radical, hydrogen peroxide, and hydroxyl radical [24]. These ROS molecules are characterized by a high reactivity as they constantly try to pair with the electrons in other molecules (e.g., protein, DNA, lipid, etc.) [4].

O_2_ + e^−^ → O_2_•^−^ (superoxide anion radical) (2)

O_2_•^−^ + e^−^ + 2H^+^ → H_2_O_2_ (hydrogen peroxide) (3)

H_2_O_2_ + e^−^ → HO• (hydroxyl radical) + OH^−^ (4)

HO• + e^−^ → OH^−^ (5)

A low concentration of ROS acts as an intracellular second messenger involved in signaling pathways [3]. Excess ROS, on the contrary, induces a redox imbalance between production and removal of ROS, and when the threshold is finally exceeded, causes damage to the cells, which is known as oxidative stress. Oxidative stress has been known as a factor causing various diseases, including cancer, diabetes, inflammation, and neurodegenerative diseases, by driving the oxidative modification of biomolecules including proteins, DNA, and lipids [4,25,26,27]. The common ROS-mediated oxidative modification of protein is the oxidation of a specific amino acid residue. ROS converts the thiol (–SH) group of a Cys residue to a sulfenic form (–SOH) that is able to create a disulfide (–S–S–) bond with sulfenic forms in other proteins. Furthermore, excess ROS influences the structure and function of proteins by generating sulfinic (–SO_2_) or sulfonic (–SO_3_) forms [28]. In a similar way, methionine is also converted to methionine sulfoxide or methionine sulfone by the action of ROS on the sulfur atom (S) [29]. Lysine, arginine, threonine, and proline residues are the amino acid residues that undergo an irreversible and irreparable carbonylation [30]. In addition to the changes in proteins, ROS causes epigenetic changes such as DNA methylation. For instance, the hypermethylation of the CpG Island in the promoter region due to ROS induces the silencing and inactivation of tumor suppressor genes to facilitate tumorigenesis in cancer [31]. ROS also induces oxidative modification of DNA, including nucleic acid mutation, double or single strand break, and base modification [32].

Cancer is a major heterogeneous disease that is closely associated with the increase in ROS in the cells and the imbalance of unstable ROS [33]. Numerous studies have shown that ROS facilitates carcinogenesis, such as proliferation, metastasis, and angiogenesis, by activating cancer signaling [34,35,36]. The transcription factor hypoxia-inducible factors (HIFs) that facilitate angiogenesis are hydroxylated by prolyl hydroxylase domains (PHDs) to be degraded by Von Hippel-Lindau (VHL), an E3 ubiquitin ligase [37]. ROS, however, eventually promotes the activation of HIFs by suppressing the activity of PHDs [38]. A form of duality is exhibited by p53, as it displays antioxidant activity in low ROS concentrations but a prooxidant activity in high ROS concentrations [39]. p53 is also involved in cell cycle arrest as one of the guardians of the genome, while participating in genome stability by binding directly to the DNA damaged by ROS. Finally, the change in downstream gene expression or the dysfunction of DNA repair causes apoptosis of cancer cells [40]. Moreover, in a normal state, the transcription factor nuclear factor (erythroid-derived 2)-like 2 (NRF2) is degraded by proteasome via an E3 ubiquitin ligase KEAP1; however, in a state of increased ROS, p21, a target gene of p53, binds to NRF2 in competition with KEAP1 to break up the KEAP1-NRF2 complex. The stabilized NRF2 enters the nucleus and binds to specific antioxidant responsive elements, thereby increasing the expression of the antioxidant genes (NQO1 and HO-1) [41]. However, p53 carries a mutation in various cancer cells, so that p21 cannot perform its normal function and cancer cell growth is increased while apoptosis is suppressed [42]. PI3K induces the phosphorylation of PIP_2_ leading to its product PIP_3_. Cancer proliferation is increased when PIP3 recruits PDK1 that induces the phosphorylation of Akt (T308) for activating the PI3K/Akt pathway [43]. Phosphatase and tensin homologue deleted on chromosome 10 (PTEN) is a type of tumor suppressor and a protein mediating the dephosphorylation of PIP3 to negatively regulate the PI3K/Akt pathway. ROS enhances the formation of PIP3 by reducing the phosphatase activity of PTEN. In addition, due to a mutation of PTEN in various cancer cells, proliferation of cancer cells increases [44,45].

## 3. Antioxidant Enzymes; SOD, CAT, GPx, and Prx

Cells have diverse defense systems to protect themselves from the oxidative stress arising from various situations and the diseases caused by them. Among such systems, the most effective method of reducing intracellular oxidative stress comes from the antioxidant enzymes that act as scavengers as the main line of defense against ROS by removing ROS through direct reduction [46]. Antioxidant enzymes are present not only in animals, but also in plants, yeasts, and bacteria, as they provide the most fundamental and essential defensive mechanism [47,48,49].

### 3.1. SOD

SOD was discovered in 1968 [6]. The enzyme removes superoxide anion (O_2_•^−^), one of the pro-oxidants (2 × 10^9^ M^−1^ sec^−1^) [50]. By facilitating the dismutation reaction of two toxic superoxide anions, SOD converts them to less toxic hydrogen peroxide, thereby protecting the cells [51].

2O_2_•^−^ + 2H^+^ → H_2_O_2_ + O_2_ (6)

As the first line of defense against ROS, SOD plays a key role in the removal of free radicals [52]. SOD is a metalloenzyme that relies on a metal cofactor for its activity. Three isoforms of SOD are found in humans. The most abundant is SOD1 (heterodimer, 32 kDa), which is present in the cytosol and nucleus, and uses copper (Cu) and zinc (Zn) as its cofactors. SOD2 (tetramer, 96 kDa) uses manganese (Mn) as its cofactor and is found in the matrix of the mitochondria. Lastly, SOD3 (tetrameric glycoprotein, 135 kDa) uses Cu and Zn as its cofactors, and as a secreted protein, it is mostly located in the extracellular matrix [53,54].

### 3.2. CAT

Hydrogen peroxide (H_2_O_2_) produced by the reduction of superoxide anion radicals are non-radical molecules with lower reactivity than superoxide anion radicals. However, since they can turn into hydroxyl radicals (OH•) via the Fenton reaction, rapid removal is required [55]. CAT was the first antioxidant enzyme discovered in 1818 and it acts to remove H_2_O_2_ [7]. A single CAT can remove approximately a million molecules of H_2_O_2_ per second so it plays a crucial role in the removal of H_2_O_2_. CAT is a tetrameric protein of 225–270 kDa [56]. The NADPH present in each subunits are used as the cofactor, whereby two H_2_O_2_ molecules are converted to water and oxygen [57].

2H_2_O_2_ → 2H_2_O + O_2_ (7)

### 3.3. GPx

GPx found in 1957 is also the first line of defense against ROS just like SOD and CAT [8]. GPx is a selenoprotein with selenocysteine (Sec, Se-Cys) as the amino acid residue, where selenium is used as the cofactor [52]. There are eight families of GPx, and H_2_O_2_ is converted to water through the action of the glutathione enzyme system (glutathione reductase (GR), glutathione (GSH), and oxidized glutathione (GSSG)) of GPx (Table 1) [58].

H_2_O_2_ + 2GSH → 2H_2_O + GSSG (8)

### 3.4. Prx

Prx is another member of the first defense that removes H_2_O_2_ such as SOD, CAT, and GPx. Prx is a thiol-specific peroxidase enzyme that is ubiquitously expressed in various intracellular organelles [60,61]. In contrast to other antioxidant enzymes (CAT or GPx), Prx does not require a cofactor when it converts H_2_O_2_ to water [10]. Since the discovery of TSA (thiol-specific antioxidant) in yeast in 1987 as the first Prx, the name has been changed from TPx (thioredoxin peroxidase) to the present Prx (peroxiredoxin). To date five Prx isoforms have been reported in yeast and six in mammalian cells [62]. Mammalian Prxs have a conserved redox-active Cys residue (peroxidatic Cys; C_P_) and a resolving Cys (C_R_), and according to the position and number of this Cys, Prx is classified into three isoforms: Typical 2-Cys (Prx1-4), atypical 2-Cys (Prx5), and 1-Cys (Prx6) (Table 2) [11].

## 4. Biological Functions of Prx1 and Prx2

### 4.1. Peroxidase Function

**Figure 1:**
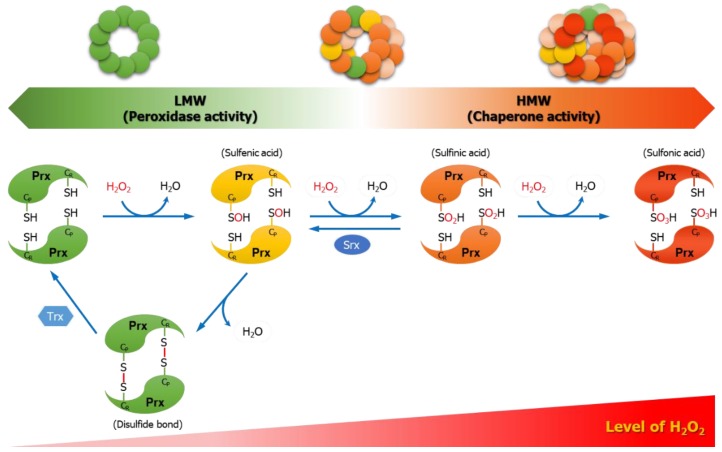
Catalytic cycle of typical 2-cysteine (Cys) Prxs. During the catalytic cycle of reducing hydrogen peroxide (H_2_O_2_) to water (H_2_O), typical 2-Cys Prxs (Prx1–4) undergo H_2_O_2_-mediated conversion of peroxidatic Cys (C_P_–SH) to sulfenic acid (C_P_–SOH) and the formation of intermolecular disulfide bond (C_P_–S–S–C_R_) with the resolving Cys (C_R_–SH) of other subunits. Prxs are then reduced back by the reducing equivalents. The hyperoxidation of the sulfenic acid (C_P_–SOH) of Prxs into sulfinic acid (C_P_–SO_2_H) occurs competitively with disulfide bond formation. The hyperoxidation of Prxs is dependent on H_2_O_2_ concentration, and the sulfinic acid of Prxs can be reversibly reduced by sulfiredoxin (Srx). However, when further hyperoxidation leads to the state of sulfonic acid (C_P_–SO_3_H), it is no longer reversible. The hyperoxidized form of Prxs loses the peroxidase activity and, with the structural changes from a low-molecular-weight (LMW) to a high-molecular-weight (HMW) complex, the chaperone activity is increased.

During the catalytic cycle of H_2_O_2_ removal, typical 2-Cys Prxs (Prx1-4) first undergo the oxidation of free thiol (C_P_–SH) in peroxidatic Cys into sulfenic acid (C_P_–SOH) intermediate. This is followed by the second reaction producing water via a resolution reaction, whereby sulfenic acid (C_P_–SOH) is locally unfolded to form an intermolecular disulfide bond (C_P_–S–S–C_R_) with the nearby resolving Cys to create a head-to-tail homodimer structure [61]. Here, atypical 2-Cys Prx5 forms an intramolecular disulfide bond, while 1-Cys Prx6 does not form a disulfide bond [61]. The Trx/TrxR system then reduces the disulfide bond for recycling as free thiol [69]. Among the six isoforms, the most abundantly expressed Prx1 and Prx2, in an adequate H_2_O_2_ level, exhibit dominant peroxidase activity that removes intracellular H_2_O_2_ through the catalytic cycle of oxidation-disulfide bond formation-reduction cycle of C_P_ residue. However, in a persistently high H_2_O_2_ level, hyperoxidation occurs to the peroxidatic Cys. The sulfenic acid (C_P_–SOH) from the oxidation by H_2_O_2_ undergoes hyperoxidation into sulfinic acid (C_P_–SO_2_H). Here, sulfinic acid (C_P_–SO_2_H) can return to sulfenic acid (C_P_–SOH) by a reversible reaction mediated by Srx. On the other hand, the sulfonylated Prx1 and Prx2 (C_P_–SO_3_H) forms are irreversible and even Srx cannot mediate their reduction. The hyperoxidized forms (C_P_–SO_2_H or C_P_–SO_3_H) of Prx1 and Prx2 lose the function of removing H_2_O_2_ as a peroxidase (Figure 1) [70].

### 4.2. Chaperone Function

In a high concentration of H_2_O_2_, a redox-dependent conformational change is induced in the structures of the hyperoxidized Prx1 and Prx2, which converts them from low-molecular-weight (LMW) to HMW complexes. At this time, chaperone activity is increased simultaneously with the decrease of peroxidase activity [16]. Typical 2-Cys Prxs have the Gly-Gly-Leu-Gly (GGLG) and Tyr-Phe (YF) motifs, which are the structural motifs acting as a floodgate that impart sensitivity to H_2_O_2_. Hence, they play a critical role in the regulation of hyperoxidation and HMW formation [71]. Prx5 and Prx6 do not have such motifs [72]. The susceptibility of Prx1 and Prx2 to H_2_O_2_ is different. Prx2 showed higher sensitivity to hyperoxidation than Prx1 did both in vitro and in vivo [73,74,75]. Mitochondrial Prx3, as in the case of Prx1 and Prx2, also has YF and GGLG motif. However, despite considerable sequence homology, it is more resistant to hyperoxidation than Prx1 or Prx2. Such variation in differential susceptibility is due to the differences in the sequence around the resolving Cys positioned at the C-terminus of Prx3 [76]. In contrast to eukaryotic Prxs that are sensitive to H_2_O_2_, prokaryotic Prxs (bacterial Prx, AhpC) lacking GGLG and C-terminal YF motif are generally resistant to hyperoxidation although there are susceptible prokaryotic Prxs. [70,77].

## 5. Interaction Partners of Prx1 and/or Prx2

Recently, others suggest that the abundant Prx1 and Prx2 act as fine-tuners by regulating the redox status, activity, and function of interaction partners through specific protein-protein interaction [78]. Furthermore, Prx1 and Prx2 participate in the oxidative modification of interaction partners via thiol-dependent/independent reaction at their active site Cys in several ways. First, the oxidized Prx dimer directly exchanges the disulfide bond with a signaling target. Second, the sulfenic acid formed first on the peroxidatic Cys of Prx forms a mixed disulfide bond with a signaling target to induce redox relay. Third, Prx allows other signaling target to be oxidized while undergoing the hyperoxidation and inactivation. Prx1 and Prx2 can facilitate redox signaling in these modes, thereby mediating the activation of various signaling pathways in cancer [69,79].

### 5.1. Interaction Partners of Prx1

#### 5.1.1. Androgen Receptor

Hypoxia is a key factor regulating tumor progression such as angiogenesis, metastasis, invasion, and proliferation, in the tumor microenvironment [80]. In prostate cancer, hypoxia activates the transcription factor androgen receptor (AR) by increasing the binding between AR and androgen responsive elements (AREs), which induces the expression of its target gene, prostate-specific antigen (PSA), and thereby regulating tumor progression [81]. Hypoxia is known to increase the expression of Prx1 [82,83]. With hypoxia, the interactions of Prx1 and AR increase, which facilitates the binding of AR to AREs that induces the expression of PSA. Here, Prx1 is oxidized by hypoxia so that its peroxidase activity is inactivated. This leads Prx1 to increase the level of AR transactivation, irrespective of its antioxidant activity. Consequently, Prx1 acts to facilitate tumor progression by increasing the transactivation of AR in prostate cancer [84].

#### 5.1.2. Apoptosis Signal-Regulating Kinase 1

**Figure 2:**
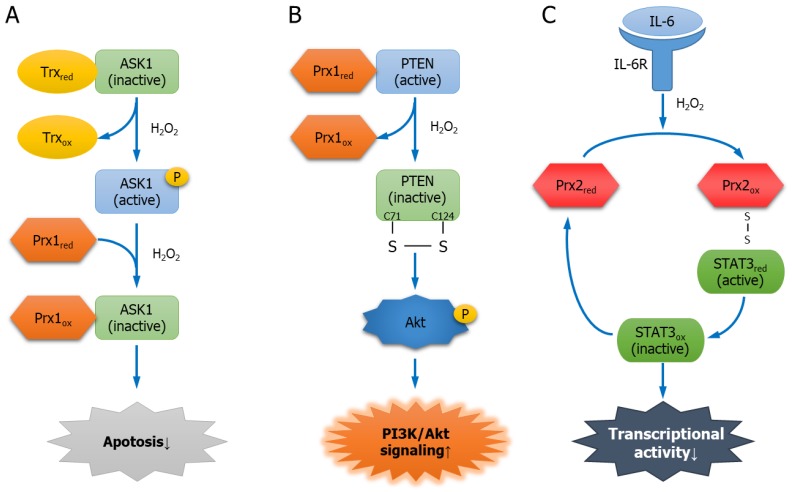
The regulatory mechanism of Prx1 or Prx2 interacting partners. (**A**) Interaction between Prx1 and apoptosis signal regulating kinase 1 (ASK1). ASK1, which induces apoptosis, is inactivated by interaction with thioredoxin (Trx). H_2_O_2_ activates ASK1 by interfering with the interaction between the two proteins. However, high concentrations of H_2_O_2_ increase the interaction between Prx1 and ASK1. As a result, Prx1 decreases ASK1 activity and inhibits apoptosis; (**B**) interaction between Prx1 and phosphatase and tensin homolog (PTEN). Prx1 interacts with PTEN to protect its lipid phosphatase activity. However, H_2_O_2_ oxidizes Prx1 and dissociates it from PTEN. PTEN is inactivated by forming a disulfide bond. Finally, Akt phosphorylation is induced and PI3K/Akt signaling is activated; (**C**) interaction between Prx2 and signal transducer and activator of transcription 3 (STAT3). Interleukin-6 (IL-6) or H_2_O_2_ oxidizes Prx2. Oxidized Prx2 binds to STAT3, causing redox relay and oxidizing STAT3 by forming disulfide exchange intermediates. The transcriptional activity of oxidized STAT3 is decreased.

Apoptosis signal-regulating kinase 1 (ASK1), a Ser/Thr kinase, induces apoptosis when it is activated [85]. In a non-stressed condition, ASK1 forms a Trx-ASK1 complex with reduced Trx, and the kinase activity of ASK1 is inhibited [86,87]. However, stressed conditions such as rotenone, tumor necrosis factor alpha (TNF-α), endoplasmic reticulum (ER) stress, and H_2_O_2_, lead to the oxidation of Trx, and the oxidized Trx dissociates from ASK1, leaving ASK1 free to be activated. Consequently, ASK1-mediated differentiation or apoptosis is induced [88,89]. Prx1 is another redox regulatory protein of ASK1 that binds through the Trx-binding domain of ASK1. The two catalytic Cys52 and Cys173 in Prx1 are crucial to the binding. When H_2_O_2_ level increases, the binding between Prx1 and ASK1 increases. As a result, the oxidized Prx1 acts as a negative regulator of ASK1, suppressing the activity of ASK1, and thus, inhibiting ASK1-induced apoptosis (Figure 2A) [17,90].

#### 5.1.3. PTEN

PTEN is a lipid phosphatase with a phosphatase domain and is a tumor suppressor protein that mediates the dephosphorylation of PIP_3_ into PIP_2_ to inhibit tumorigenesis via PI3K/Akt signaling [44]. Prx1 interacts with PTEN, while the two Cys residues (Cys52 and Cys173) of Prx1 play a crucial role in protecting the lipid phosphatase activity of PTEN and inhibiting Ras-induced transformation. However, an increase in H_2_O_2_ causes hyperoxidation of Prx1 to break up its binding with PTEN. The dissociated PTEN undergoes oxidation so that Cys71 and Cys124 in the phosphatase domain forms a disulfide bond to cause inactivation. Consequently, the inactivation of PTEN induces the phosphorylation of the suppressed Akt and promotes tumorigenesis through H-Ras and ErbB-2-induced transformation (Figure 2B) [91,92].

#### 5.1.4. Mammalian Ste20-like Kinase-1

Mammalian Ste20-like kinase-1 (MST1), a Ser/Thr kinase, is a tumor-suppressor protein whose kinase activity is activated by H_2_O_2_ [93,94]. Prx1 is oligomerized to increase the association with MST1 depending H_2_O_2_ stimulation in its binding to MST1, which enhances the activity of MST1. In addition, mutations of Cys52 and Cys83 in Prx1 inhibit the H_2_O_2_-induced activation of MST1, whereas Cys173 mutation promotes the activation of MST1, which makes the Cys residue of Prx1 invaluable in MST1 activation. In contrast, Prx2, a homologue of Prx1, does not bind to MST1. Consequently, the activation of MST1 by Prx1 led to H_2_O_2_-induced cancer cell death [95].

#### 5.1.5. Glycerophosphodiester Phosphodiesterase 2

Glycerophosphodiester phosphodiesterase 2 (GDE2) is a six-transmembrane protein involved in the progression of motor neuron differentiation in the spinal cord, which is required in motor neuron generation [96]. GDE2 exists in the membrane in an inactivated state due to the intra-disulfide bond between Cys25 of the N-terminal domain and Cys576 of the C-terminal domain. The interaction of reduced Prx1 with GDE2 results in the reduction of the disulfide bond in GDE2 while Prx1 is oxidized. The oxidized Prx1 loses the interaction with GDE2, making it undergo a redox-dependent activation, which facilitates the motor neuron differentiation [18].

#### 5.1.6. Apurinic/Apyrimidinic Endonuclease 1

Apurinic/apyrimidinic endonuclease 1 (APE1) is an AP endonuclease with nucleotide incision repair activity that repairs DNA lesions damaged by ROS [97]. In addition, APE1 is a redox factor that plays a role in maintaining the reduced state of various transcription factors such as NF-κB, HIF, or p53 [98]. Prx1 binds to APE1 and blocks its redox activity, inhibiting APE1/NF-κB-dependent pro-inflammatory chemokine IL-8, thereby inhibiting the migration, invasion, and metastasis of inflammation-associated cancer cells such as in gastric cancer. On the contrary, E3330, a specific inhibitor that binds to APE1 and inhibits the redox activity of APE1, induces the dissociation of heterodimeric complex between Prx1 and APE1 [99].

### 5.2. Interaction Partners of Prx2

#### 5.2.1. Platelet-Derived Growth Factor Receptor

Platelet-derived growth factor (PDGF) induces the phosphorylation of PDGF receptor (PDGFR) to increase the activation of various downstream molecules, including phosphatidylinositol 3′-kinase (PI3K) and phospholipase C-γ1 (PLC-γ1), whereby cellular signaling is activated [100,101,102]. In addition, PDGF induces the increase in intracellular H_2_O_2_ [103]. Under a PDGF stimulation condition, Prx2 not only inhibits the production of H_2_O_2_, but also reduces the protein tyrosine phosphatase (PTPase) activity by binding to the H_2_O_2_-activated PDGFR. In addition, Prx2 inhibits the phosphorylation of PLC-γ1. On the other hand, when Cys residues of Prx2 are mutated (C51/172S), PDGFR activity is not affected so that peroxidase activity of Prx2 is required for PDGF signaling. Prx1, in contrast to Prx2, does not interact with PDGFR. Consequently, Prx2 acts as a negative regulator of PDGF signaling to inhibit the proliferation and migration of smooth muscle cells [104].

#### 5.2.2. Tankyrase

β-catenin exhibits an oncogenic function because of the increased expression or mutation occurring in various cancers. Axin, a scaffold protein, recruits adenomatous polyposis coli (APC), casein kinase1 (CK1), and Ser/Thr kinases GSK3, to form a “β-catenin destruction complex” [105]. β-catenin is degraded by proteasome when GSK3-mediated phosphorylation leads to its ubiquitination by E3-ubiquitin ligase β-TrCP. In addition, Axin mediates the phosphorylation on Ser45 of β-catenin by CK1, thereby facilitating the degradation of β-catenin [106,107]. Tankyrase (TNKS), as a member of the poly(ADP-ribose) polymerase (PARP) superfamily, mediates the poly-ADP-ribosylation (PARylation) of its target protein. PARylation is a post-translational modification that creates a poly(ADP-ribose) chain by continuously combining the target protein to ADP-ribose using β-NAD^+^ as the substrate [108,109]. TNKS binds to Axin to induce its PARylation, and the PARylated Axin undergoes ubiquitination for the degradation by proteasome [110]. APC that composes the β-catenin destruction complex is a tumor suppressor and shows a mutation in over 50% of colorectal cancer (CRC) patients. The mutation in APC increases the level of intracellular H_2_O_2_ to suppress the activity of TNKS. Prx2, however, removes H_2_O_2_ by binding to TNKS, and hence TNKS retains its activity to degrade Axin, leading to the increase in β-catenin. The resulting increase in β-catenin allows it to enter the nucleus where it binds to the transcription factor TCF/LEF for the expression of the target gene, thus facilitating tumorigenesis. In the absence of Prx2 or in the case of Prx2 Cys mutant (C51/172S), the lack of binding between Prx2 and TNKS leads to the increase in H_2_O_2_ and TNKS is inactivated. As a result, Axin is stabilized, thereby mediating the degradation of β-catenin. Consequently, Prx2 plays a role in promoting the tumorigenesis in CRC [111].

#### 5.2.3. Signal Transducer and Activator of Transcription 3

Cytokine Interleukin-6 (IL-6) binds to the IL-6 receptor (IL-6R), resulting in JAK phosphorylation. The activated JAK mediates the phosphorylation of the transcription factor signal transducer and activator of transcription 3 (STAT3). The phosphorylated STAT3 enters the nucleus, where it suppresses apoptosis or facilitates tumorigenesis [112]. H_2_O_2_ stimulates the JAK/STAT pathway [113]. Four Cys residues (Cys866, Cys917, Cys1094, and Cys1105) of JAK2 are critically involved in its catalytic activity [114]. H_2_O_2_ oxidizes Prx2, and the oxidized Prx2 forms disulfide exchange intermediates based on STAT3 and disulfide-linked conjugate, which prompts the redox relay that oxidizes STAT3. The formation of a disulfide-linked STAT3 oligomer through the redox relay reduces the transcriptional activity of STAT3. The oxidation of STAT3 is inhibited when Prx2 level is decreased or its Cys residues are mutated (C51/172S). In addition to H_2_O_2_, IL-6 oxidizes Prx2, and Prx2 reduces the cytokine-induced STAT3-mediated transcriptional activity. Consequently, Prx2, together with STAT3, regulates the transcriptional activity of STAT3 via redox relay (Figure 2C) [115].

## 6. Conclusions

Prxs are peroxidases that remove H_2_O_2_ through thiol-dependent oxidation, which distinguishes them from other antioxidant enzymes that scavenge H_2_O_2_. Among Prxs, the most well-known ones are cytosolic Prx1 and Prx2 that not only protect the cells, but also regulate redox signaling by removing H_2_O_2_ using the two conserved Cys residues that are essential in peroxidase activity. Furthermore, Prx1 and Prx2 are multifunctional proteins whose function may be switched to a chaperone activity when H_2_O_2_ causes their hyperoxidation. Among the non-catalytic Cys residues of Prx1, Ser83 induces structural and functional changes, which implies the need for studies on examining whether the Cys residues—71 in Prx1 and 70 in Prx2—bring about changes to the peroxidase or chaperone activity [116]. Further studies are also required for investigating the role of such non-catalytic Cys in redox signaling. Increasing number of studies reported on the role of the abundant Prx1 and Prx2 in the regulation of the redox state, activity, or function of the target protein based on the disulfide bond interaction with other proteins. Thus, it is deemed necessary that an in-depth study should focus on the mechanisms of Prx1 or Prx2 in the light of the signaling pathways regulated by them, as well as the functions of the proteins interacting with Prxs in the cells.

## Figures and Tables

**Table 1:** Glutathione peroxidase (GPx) isoforms in mammalian cells.

Isoform	Localization	Catalytic Residue	Ref.
GPX1	Cytosolic	Sec	[52,59]
GPX2	Gastrointestinal	Sec	[52,59]
GPX3	Plasma	Sec	[52,59]
GPX4	PH ^1^	Sec	[52,59]
GPX5	EL ^2^	Cys	[52,59]
GPX6	Olfactory	Sec	[52,59]
GPX7	ER ^3^	Cys	[52,59]
GPX8	ER ^3^ (putative)	Cys	[52,59]

^1^ PH: Phospholipid hydroperoxide. ^2^ EL: Epididymal lumen. ^3^ ER: Endoplasmic reticulum.

**Table 2:** Peroxiredoxin (Prx) isoforms in mammalian cells.

Class	Isoform	C_P_	C_R_	Non-Catalytic	Localization	Ref.
Typical 2-Cys	Prx1	52	173	71, 83	Cytosol, nucleus, PM ^1^	[11,63]
	Prx2	51	172	70	Cytosol, nucleus, PM ^1^	[11,64]
	Prx3	108	229	127	Mitochondria	[11,65]
	Prx4	87	208	14, 111	Cytosol, secretion, ER ^2^	[11,66]
Atypical 2-Cys	Prx5	47	151	72	Cytosol, peroxisome mitochondria	[11,67]
1-Cys	Prx6	47	−	91	Cytosol, secretion lysosome	[11,68]

^1^ PM: Plasma membrane. ^2^ ER: Endoplasmic reticulum.
